# Clinical Microbiological Aspects of Epileptic Seizures in the Tropical Countries with Specific Focus on Nigeria

**DOI:** 10.1100/tsw.2005.51

**Published:** 2005-05-13

**Authors:** Ijeoma Kanu, Ebere C. Anyanwu, Nkechi C. Nwachukwu, John E. Ehiri, Joav Merrick

**Affiliations:** ^1^ Department of Microbiology, Abia State University, PMB 2000, Uturu, Nigeria; ^2^ Cahers Neurosciences Research, Inc., 8787 Shenandoah Park Drive, Suite 122, Conroe, Houston, TX 77385, USA; ^3^ Department of Maternal and Child Health, School of Public Health, University of Alabama at Birmingham (UAB), UK; ^4^ National Institute of Child Health and Human Development and Center for Multidisciplinary Research in Aging, Faculty of Health Sciences, Ben Gurion University of the Negev and Office of the Medical Director, Division for Mental Retardation, Ministry of Social Affairs, Jerusalem, Israel

**Keywords:** epileptic seizures, etiology, protozoa, bacteria, fungus, virus, infections, Nigeria

## Abstract

Epilepsy is a common neurological disorder; however, in Nigeria and other tropical regions, the causes of epileptic seizures differ greatly in etiology. This paper is an attempt to highlight some possible microbiological aspects of epileptic seizures. A literature review was carried out to identify the extent to which microbial infections were involved in the elicitation of epileptic seizures. Data were collected from several clinics in the community and hospitals in Nigeria and correlated with the evidence from the literature review. It was found that different microbial agents including viral, bacterial, protozoa, and fungal agents were involved in several aspects of epileptic seizures. Malaria was found to cause more than 88% of childhood epileptic seizures and 12% of adult seizures. Generalized tonic-clonic seizures occurred in more than 40% of adult patients. Partial seizures were uncommon. Cases of epileptic seizures associated with bacteria (e.g., brucellosis), viral, fungal, and protozoa infections were frequently reported. Malaria, tapeworm, and cysticercosis were some of the common infectious causes of epilepsy; however, in some cases, the cause remained unknown. From these findings, it was evident that microbiological aspects of epilepsies are possible research areas that might be developed. It is believed that the unraveling of the various microbiological factors in epileptic seizures would have important implications for understanding the underlying neurobiology, evaluating treatment strategies, and perhaps planning health-care resources for the affected. It will also help to improve the prognostic factors in initial seizure symptomatic etiology and presence of any structural cerebral abnormalities.

## INTRODUCTION

In the course of our research in environmental microbiology, it was observed that exposures to certain environmental microbiological contaminants could lead to certain epileptic seizures in children and adolescents. The microscopic nature of the brain architecture requires that in-depth investigation be carried out to unravel how environmental exposures to chemical and microbiological contaminants lead to certain types of epileptic seizures. We have spent over 2 years in the development of an understanding of this new phenomenon in epilepsy, which we strongly believe would lead to better understanding of the microbiological aspects of epileptic seizures. Generally, seizures are a result of abnormal firing of the neurons in uncontrollable fashion, however, the term “epilepsy” refers to the tendency to experience recurrent seizures.

Epilepsy is also defined as a neurological condition, which is characterized by recurrent unprovoked seizures. Epilepsy may be idiopathic, cryptogenic, or symptomatic. Idiopathic epilepsies are generally genetic, and advances in molecular genetics will undoubtedly reveal many more syndromes in the near future[1,2,3,4,5,6,7,8]. Cryptogenic epilepsies are those in which an underlying cause is suspected, but the etiology remains undetected[1]. Epilepsies for which there is an underlying structural cause or major metabolic derangement are considered symptomatic[1]. The clinical manifestation of epileptic seizures may vary widely from patient to patient, depending on the region of the brain involved. Over the centuries, many seizure classification systems have been used and the current, most widely used classification system is that of the International League Against Epilepsy (ILAE). The ILAE system divides seizures into those of partial onset and those of generalized onset, depending on whether the initial clinical manifestations indicate that one cortical region or both hemispheres are involved at the onset of the seizure[1]. Partial seizures are then divided into simple partial seizures, in which a fully conscious state is retained, or complex partial seizures, in which consciousness is impaired[7,9,10,11,12]. Seizures that are secondary to CNS infections are among the most refractory medically and thus insult-specific agents are desirable. Recently, the study of the neuropharmacological actions of dynorphin in CNS viral injury has yielded new insights into epileptogenesis and epilepsy treatment[3]. Hence, there is an interest in the role of dysregulation of dynorphin in the pathogenesis of refractory seizures, including encephalitic seizures as evidenced in the new models of symptomatic epilepsies based on viral-induced seizures[3].

This research report is aimed at reviewing some of the pieces of evidence that support the need for further development of the microbiological aspects of epilepsies. It is believed that the unraveling of the various microbiological factors in epileptic seizures would have important implications for understanding the underlying neurobiology, evaluating treatment strategies, and perhaps planning health-care resources for the affected. It will also help to improve the prognostic factors in initial seizure symptomatic etiology and presence of any structural cerebral abnormalities[2,10,12].

## METHODS

Extensive literature reviews on the topic were first carried out to identify the extent to which microbial infections were involved in the elicitation of epileptic seizures. Then, data were collected from ten different private community clinics and hospitals using specially designed questionnaires involving the demography, child and adult distribution, and the microbial diagnostic outcomes of the epileptic seizure cases seen during the period 2002 to 2004, inclusive. The data collected were tabulated and used to compare and correlate with the evidence from the literature reviews.

## RESULTS

Our study comprised 496 epileptic seizure cases. The age range of all the cases was between 2 and 64 years; 273 (55%) were females, while 223 (45%) were males. Details of the findings are presented in

Tables 1 and 2. Malaria was found to cause more than 88% of childhood epileptic seizures (mostly convulsions) and 12% of adult seizures.

**TABLE 1**
**Clinical Details, Adult and Child Distribution of Different Microbial Causes of Epileptic Seizures**

| Clinical Details | | | | Causes | | | | |
|---|---|---|---|---|---|---|---|---|
| **Clin./ Hosp. No.** | **No. of Cases** | **Adult (>19 Years)/%** | **Children (<20 Years)/%** | **Malaria** | **Tapeworm** | **Viral/ Bacterial** | **Cysti- cercosis** | **Unknown** |
| 1 | 32 | 4 | 28 | 21 | 3 | 5 | 2 | 1 |
| 2 | 15 | 2 | 13 | 10 | 2 | 2 | 1 | 1 |
| 3 | 112 | 20 | 92 | 73 | 11 | 17 | 8 | 3 |
| 4 | 17 | 2 | 15 | 11 | 2 | 3 | 1 | 1 |
| 5 | 21 | 3 | 18 | 14 | 2 | 3 | 2 | 1 |
| 6 | 42 | 4 | 38 | 27 | 4 | 6 | 3 | 1 |
| 7 | 81 | 8 | 73 | 53 | 8 | 12 | 6 | 2 |
| 8 | 102 | 11 | 91 | 66 | 10 | 15 | 7 | 3 |
| 9 | 12 | 2 | 10 | 8 | 1 | 2 | 1 | 1 |
| 10 | 62 | 5 | 57 | 40 | 6 | 9 | 4 | 2 |
| **Total** | **496** | **61** | **435** | **323** | **49** | **74** | **35** | **16** |

**TABLE 2**
**Percentage Distribution of Microbial Causes**

| Causes | No. | % |
|---|---|---|
| Malaria | 323 | 65 |
| Worms | 49 | 10 |
| Virus | 74 | 15 |
| Cysticercosis | 35 | 7 |
| Unknown | 16 | 3 |

Other causes were parasitic worms especially tapeworms (10%), viral/bacteria (15%), cysticercosis (7%), and 3% were of unkown cause (see Table 2). Generalized tonic-clonic seizures occurred in more than 40% of adult patients. Partial seizures were uncommon. Cases of epileptic seizures associated with bacteria (e.g., brucellosis), viral, fungal, and protozoa infections were frequently reported. Malaria, tapeworm, and cysticercosis are some of the common infectious causes of epilepsy; however, in some cases, the cause remained unknown.

Overall, it was found that different microbial agents including viral, bacterial, protozoa, but no fungal, agents were involved at least in several aspects of epileptic seizures as supported by pieces of evidence from peer-reviewed literatures and the clinic data obtained.

## DISCUSSION

In Nigeria, it was observed that bacterial infections are often the cause of certain types of epileptic seizures in children; seizures that occur before or on diagnosis only (type I seizures) and seizures that occur before and/or after diagnosis (type II seizures). Deaths usually occur in 14/34 and 11/34 children with type I and type II seizures, respectively ($p < 0.0001$)[4]. Angyo et al.[5] performed blood cultures on 891 children (aged 1 month to 16 years), who presented at the Jos University Teaching Hospital (JUTH) with presumed diagnosis of septicemia, over a 3-year period (January 1994 to December 1996) in order to determine the predominant bacterial agents. Out of 891 blood cultures, 139 (15.6%) were positive. Five of the blood cultures yielded double isolates. *Staphylococcus aureus* was the most common bacterial agent isolated and accounted for 36% of all the isolates. *Klebsiella* and *Salmonella* spp. each accounted for 18.7 and 15.8%, respectively. Other bacterial agents isolated included *Escherichia coli,* 7.9%; coliforms (untyped), 6.5%; *Pseudomonas* spp., 3.6%; *Proteus* spp., 2.2%; and miscellaneous organisms, 9.3%.

*Salmonella enterica* ssp. *houtenae* infection was shown to lead to focal epileptic seizures and multiple brain abscesses as revealed by brain resonance imaging, and electroencephalogram showed epileptiform activity[6]. Consequently, it was reported that intravenous antimicrobial agents for a total of 84 days and interferon-gamma were able to cure the affected without any neurologic sequel[6]. The incidence of viral infections in the CNS of neonates with seizures of unknown origin may be much higher than previously reported[7,16]. Convulsions are common complications of shigellosis in children[8,17]. Other infected individuals may show refractory status epilepticus, shock[9,18], and epileptic seizures revealing a menses toxic shock syndrome have also been reported[10].

### Microbiological Aspects of Cryptogenic Epilepsy

Cryptogenic epilepsy, the group of epilepsy syndromes for which an etiology is unknown, comprises approximately 20% of all epilepsy syndromes[1,11]. Stommel et al.[30] selected patients in this subgroup of epilepsy and tested them for evidence of *Toxoplasma gondii* IgG antibodies by the enzyme-linked immunosorbent assay. *T. gondii* was found in up to 20% of the U.S. population forming dormant brain cysts in the latent bradyzoite form. We have found a statistically significant elevation of *T. gondii* antibodies among cryptogenic epilepsy patients as compared to controls (59% increase in optical density [OD], $p = 0.013$). This association persisted after adjustment for subjects' gender and age in a multiple logistic regression model. However, it was no longer as statistically significant suggesting that chronic *T. gondii* infection with brain cysts may be a cause of cryptogenic epilepsy[11,30]. In Chulalongkorn Hospital, Bangkok, Thailand for example, approximately 70% of the children developed epileptic seizures caused by upper respiratory tract infection, nonspecific febrile illness, diarrhea, urinary tract infection, viral infection, pneumonia, herpangina, measles, pneumococcal bacteremia, and dengue fever[12,31]. Single epileptic seizures have been identified as isolated neurological complication of mycoplasma pneumoniae infection[13,32]. Juntunen et al.[33] reported the cases of a group of 22 previously healthy children with their first convulsive status epilepticus (SE), treated at Kuopio University Hospital, Finland, prospectively studied. Eleven of the 22 children had febrile SE, where viral infection was identified in 10 of the 11 febrile children. Human herpes virus 6 infection was identified in 12 children (55% and at least 6 of them as primary infection). Single cases of human herpes virus 7, parainfluenza 3, adenovirus 1, echovirus 22, rota, influenza A and *Mycoplasma pneumoniae* infection were diagnosed and they therefore concluded that virus (human herpes virus 6 in particular) seemed to be associated with convulsive SE, both febrile and afebrile. Human herpes virus 7 and *M. pneumoniae* are new agents associated with SE[14,33,34].

It is also suggested that the causative agents of febrile illness associated with seizures in summer season are primarily enteroviruses, especially coxsackieviruses group A and that febrile seizures might be caused by enteroviral infection in the central nervous system[15,16,17,18,19,34]. Alterations in consciousness, including seizures, delirium, and coma are known to occur during *Shigella* infection.

Previous reports have suggested that febrile convulsions and altered consciousness are more common during shigellosis than with other childhood infections[16].

## Involvement of Viral Infection in Epilepsies

Epileptic seizures are frequently seen after viral encephalitis. Herpes simplex virus type 1 (HSV-1) encephalitis is the most common cause of acquired epilepsy in humans[17,35]. HSV-1 infection induces epileptiform activity, neuron loss, and subsequently, a dramatic increase of mossy fiber sprouting in the supragranular area. With intracellular recordings, surviving CA3 pyramidal cells exhibited a more depolarizing resting membrane potential concomitant with an increase in membrane input resistance and had a lower threshold to generate synchronized bursts and a decrease in the amplitude of afterhyperpolarization than did controls suggesting that a direct change in the excitability of the hippocampal CA3 neuronal network and HSV-1–induced neuron loss resulting in subsequent mossy fiber reorganization may play an important role in the generation of epileptiform activity[17,18,35,36].

Although most human cases of West Nile (WN) fever are benign, approximately 1% produces severe neurological illness. Occipital lobe seizures, often mimicking other primary seizure types due to extraoccipital spread, are uncommon in adults and especially so from an infectious origin[19,35,36,37]. However, it is now reported that WN infections can lead to a simple partial seizure, focal motor, resulting from an occipital epileptogenic focus indicating also that the frequency of seizures in WN encephalitis virus is particularly due, in part, to cortical neuronal networks dysfunction[19]. Human herpes virus 6 and 7 infections are often related to the occurrence of febrile seizures (FS), which may be caused, in part, by their direct invasion to the CNS[15,20,38]. Also, human parvovirus B19 infections in children are more commonly associated with FS and meningoencephalitis demonstrating that, rarely, it may be associated with the development of partial epilepsy[21,39]. Herpes encephalitis, an infection of the brain itself, causes seizures in a high percentage of persons with the disease. HSV-1 is an important pathogen related to epilepsy and it has been shown that corneal inoculation of mice with HSV-1 causes acute spontaneous behavioral and electrophysiological seizures and increases hippocampal excitability and kainite-induced seizure susceptibility[22,23,24,25,43,44,45,46].

## Involvement of Malaria in Some Aspects of Epilepsies

*Plasmodium falciparum* is the most common cause of severe and life-threatening malaria. Falciparum malaria causes over one million deaths every year. In Africa, a vast majority of these deaths occur in children under 5 years of age[24,25,26,27,28,29,30]. The presentation of severe malaria varies with age and geographical distribution. The mortality rate is higher in adults than in children, but African children develop neurocognitive complications following severe malaria more frequently[24,46,47,48]. In children, the complications include metabolic acidosis (often caused by hypovolemia), hypoglycemia, hyperlacticacidemia, severe anemia, and raised intracranial pressure. Each of these conditions probably has the propensity to cause epileptic seizures[45]. In contrast, concomitant bacterial infections occur more frequently in children and are associated with mortality in children. Admission to critical or intensive care units may help reduce the mortality and the frequency and severity of complications related to severe malaria[24].

In Tanzania, the prevalence of active epilepsy was 10.2 in 1,000. Prevalence among villages varied, ranging from 5.1–37.1 in 1,000 (age-adjusted 5.8–37). In a 10-year period (1979–1988), 122 subjects living in the 11 villages developed epilepsy, with an annual incidence of 73.3 in 100,000[25]. Generalized tonic-clonic seizures accounted for 58% and partial seizures accounted for 31.9%, whereas in 10.1% seizures were unclassifiable. Of the partial seizures, secondarily generalized seizures were the most common. Possible etiologic or associated factors were identifiable in only 25.3% of cases. Febrile convulsions were associated in 13.4 of cases. Other associated factors included unspecified encephalitis (4.7%), cerebral malaria (1.9%)[46]. In Ethiopia, 139 incident cases were identified, corresponding to an annual incidence of 64 in 100,000 inhabitants (95% confidence interval [CI 44–84]). The corresponding rate for males was 72 (CI 42–102); for females, it was 57 (CI 31–84). The highest age-specific incidence occurred in the youngest age groups (0–9 years); the next highest was in the group aged 10–19 years. Generalized convulsive seizures occurred in 69%, partial seizures occurred in 20%, and unclassifiable seizures occurred in 11%. Seizures occurred daily in 10% and weekly in another 14%; 33% had monthly seizures[47]. The incidence of epilepsy in Ethiopia was high, but comparable to that in the rest of the tropical countries of the world.

Febrile seizures are the most common form of childhood seizures, occurring in 2–5% of children in the U.S. Most FS are considered simple, although those with focal onset, prolonged duration, or that occur more than once within the same febrile illness are considered complex. Risk factors for a first FS, recurrence of FS, and development of future epilepsy are identifiable and varied. Children with FS encounter little risk of mortality and morbidity and have no association with any detectable brain damage[27,28,29,30,31,32,33,34,35,36,49,50,51]. Recurrence is possible, but only a small minority will go on to develop epilepsy[27,28,29,30,31,32,33,51]. Cerebral malaria (CM) is a complication of *P. falciparum* infection in humans and is characterized by a reversible encephalopathy with seizures and loss of consciousness. Central clinical signs are partly due to sequestration of parasitized red blood cells in the brain microvasculature due to interactions between parasite proteins and adhesion molecules[28,37,38,39,40,41,42,49]. At Chikankata Hospital in rural Zambia, seizures comprised 44% of all inpatients presenting with neurological diseases and among these, children with FS had higher malarial parasite counts and required longer hospitalizations[50]. The association between epileptic seizures and malaria was significant and it was found that epilepsy and FS were responsible for a significant burden of disease in rural Zambia[29,50].

Gendrel et al.[14] evaluated the predictive value of parasitemia based on hospital admission in 69 Gabonese children aged from 3–13 years hospitalized for *P. falciparum* malarial attacks. Of these children, 14 had CM, 5 had isolated convulsions, and 50 had uncomplicated attacks. The parasitemia values measured were compared with those found in asymptomatic children of the same age range carrying trophozoites. There were no significant differences in mean parasitemia count between the three types of malarial attack and only the asymptomatic carriers had significantly lower counts. However, the wide scattering of individual values within each group indicated that simple measurement of parasitemia is not discriminative enough to predict the course of malarial attacks in children living in endemic regions. In particular, malarial attacks with very low or very high parasitemia value are possible, but similar values are found in asymptomatic carriers. The neurological complications of malaria (an almost universal problem although it occurs mainly in the tropics) constitute the most common cause of death and morbidity[51,52,53,54,55,56,57,58,59,60]. These complications usually occur in *P. falciparum* infections and in those who are nonimmune, such as children, pregnant women, and visitors to areas endemic for malaria. They are rare in children suffering from protein-calorie malnutrition. Febrile convulsions, the most common childhood emergency in Africa, and CM are the most common neurological complications. It has been suggested that various forms of mental illness and psychoses may be related to malarial infections, but research is needed to establish cause and effect relationship[14,51,52,53,54,55,56,57,58,59,60,61,62,63,64,65].

## CONCLUSIONS

The microbiological aspects of epileptic seizures have been reviewed. It was found that the microbiological aspects of epileptic seizures were under-reported, under-recognized, and under-treated. The unraveling of the various microbiological factors in epileptic seizures would have important implications for understanding the underlying neurobiology, evaluating treatment strategies, and perhaps planning health-care resources for the affected. It will help to improve the prognostic factors in initial seizure symptomatic etiology and presence of structural cerebral abnormalities. It is concluded that this area of epilepsy could hold the key to declassification of the “epilepsies of unknown etiology.” Further research in this area is ongoing and similar research pursuits are highly recommended.

## BIOSKETCHES

**Ijeoma Kanu, MSc,** is a lecturer in the Department of Microbiology, Abia State University and a PhD research student in environmental microbiology at the Michael Okpara University of Agriculture, Umudike, Umuahia, Abia State, Nigeria. E-mail: ijay2001kay@yahoo.com

**Ebere C. Anyanwu, PhD,** is a clinical neurophysiologist and director of Cahers Neuroscience Research Inc., Houston and an adjunct professor of anatomy and physiology at the North Harris and Montgomery Colleges in Houston and The Woodlands, respectively. He is also a researcher at the Biomarkers Research Laboratory, Department of Chemistry and Environmental Toxicology, Texas Southern University, Houston. E-mail: ebereanyanwu@msn.com

**Nkechi C. Nwachukwu, PhD,** is a lecturer in medical microbiology in the Department of Microbiology, Abia State University, Abia State, Nigeria. E-mail: nkinwachukwu@yahoo.com

**John Ehiri, PhD, MPH,** is an assistant professor in the Department of Maternal and Child Health, School of Public Health, University of Alabama at Birmingham (UAB), Birmingham. E-mail: jehiri@uab.edu.

**Joav Merrick, MD, DMSc,** is professor of child health and human development affiliated with the Center for Multidisciplinary Research in Aging, Zusman Child Development Center, Division of Pediatrics and Community Health at the Ben Gurion University, Beer-Sheva, Israel; the medical director of the Division for Mental Retardation, Ministry of Social Affairs, Jerusalem; and founder and director of the National Institute of Child Health and Human Development. He has numerous publications in the field of child health and human development, rehabilitation, intellectual disability, disability, health, welfare, abuse, advocacy, quality of life and prevention. Dr. Merrick received the Peter Sabroe Child Award for outstanding work on behalf of Danish Children in 1985 and the International LEGO-Prize ("The Children's Nobel Prize") for an extraordinary contribution towards improvement in child welfare and well being in 1987. E-mail: jmerrick@internet-zahav.net. Website: www.nichd-israel.com
